# PD-L1 expression in perihilar and intrahepatic cholangiocarcinoma

**DOI:** 10.18632/oncotarget.15602

**Published:** 2017-02-21

**Authors:** Jacqueline Fontugne, Jérémy Augustin, Anaïs Pujals, Philippe Compagnon, Benoit Rousseau, Alain Luciani, Christophe Tournigand, Daniel Cherqui, Daniel Azoulay, Jean-Michel Pawlotsky, Julien Calderaro

**Affiliations:** ^1^ AP-HP, Groupe Hospitalier Henri Mondor, Département de Pathologie, Créteil, France; ^2^ INSERM, U955, Team 18, Institut Mondor de Recherche Biomédicale, Créteil, France; ^3^ Université Paris Est Créteil, Créteil, France; ^4^ AP-HP, Groupe Hospitalier Henri Mondor, Département de Chirurgie Hépato-Biliaire et Transplantation Hépatique, Créteil, France; ^5^ AP-HP, Groupe Hospitalier Henri Mondor, Département d’Oncologie Médicale, Créteil, France; ^6^ AP-HP, Groupe Hospitalier Henri Mondor, Département d’Imagerie Médicale, Créteil, France; ^7^ AP-HP, Centre Hépatobiliaire, Service de Chirurgie Hépatobiliaire, Hopital Paul Brousse, Créteil, France; ^8^ AP-HP, Groupe Hospitalier Henri Mondor, Service de Virologie, Bactériologie-Hygiène, Mycologie-Parasitologie et Unité Transversale de Traitement des Infections, Créteil, France

**Keywords:** cholangiocarcinoma, PD-1, PD-L1, immunotherapy

## Abstract

Cholangiocarcinoma is an aggressive biliary neoplasm lacking effective therapeutic agents. Immunotherapies targeting the PD-L1/PD-1 immune checkpoint have shown encouraging results in solid and hematologic cancers in clinical trials. Response to these immunomodulators is correlated with PD-L1 expression. Our goal was to characterize PD-L1 expression in intra-hepatic (iCCA) and perihilar (pCCA) cholangiocarcinomas, and to correlate our results with clinicopathological features, density of tumor-infiltrating lymphocytes (TILs) and PD-1 expression.

A series of 58 iCCAs and 41 pCCAs was included in the study. PD-L1, PD-1 and CD3 expression was investigated using immunohistochemistry. Density of TILs was evaluated by immunohistochemistry using a quantitative score of CD3-stained intratumoral lymphocytes.

PD-L1 expression by neoplastic cells was observed in 9 cases (9%, 5 iCCAs and 4 pCCAs). PD-L1 positive inflammatory cell aggregates were identified in 46% (*n* = 46) of the cases (31 iCCAs and 15 pCCAs). PD-L1 expression by either neoplastic or inflammatory cells was associated to high density of CD3-positive TILs (*p* = 0.01 and *p* = 0.005, respectively). The number of PD-L1 positive inflammatory cell aggregates was higher in tumors with high PD-1 expression (*p* < 0.0001).

Altogether, PD-L1 in iCCA and pCCA is mainly expressed in tumors with high density of TILs. Our results suggest that CCAs with dense intratumoral lymphocytic infiltration might represent good candidates for PD-L1/PD-1 blocking agents.

## INTRODUCTION

Cholangiocarcinomas represent a heterogeneous group of cancers derived from epithelial biliary cells. They are classified, according to their anatomic localization on the biliary tree, into intra-hepatic (iCCA), perihilar (pCCA) or distal cholangiocarcinomas [1]. Cholangiocarcinomas are characterized by high-level resistance to conventional anticancer agents and poor clinical outcome [2]. Surgical resection, the only curative treatment, is hampered by a high rate of tumor recurrence and a low overall 5-year survival rate [3]. New treatment strategies for cholangiocarcinoma therefore represent an urgent unmet need.

Recently, clinical trials using monoclonal antibodies targeting immune checkpoint regulators, such as Programmed-death-1 (PD-1) or its major ligand, Programmed-death Ligand 1 (PD-L1), showed promising results in various hematologic and solid cancers [4]. An objective response or prolonged disease stabilization were obtained in patients with advanced melanoma, bladder urothelial carcinoma, Hodgkin's lymphoma and renal cell, colorectal or non small-cell lung carcinomas [5–10].

Under physiological conditions, PD-L1 binding to the T-cell surface receptor PD-1 induces a down-regulation of T-cell activation and signaling, as well as T-cell apoptosis, thereby suppressing auto-immunity [11, 12]. PD-L1 protein can be expressed at the surface of macrophages and lymphocytes, but this is rarely the case in non-neoplastic epithelial tissues [13, 14]. It has however been demonstrated that cancer cells can express PD-L1 to suppress the host's anti-tumor immune response and escape it [11, 13].

Multiple studies have sought to identify predictive markers of tumor response in order to select patients who would benefit most from immune checkpoint inhibitors [7, 15, 16]. Interestingly, tumor response to anti PD-L1 immunotherapies was reported to be strongly related to the tumoral expression of PD-L1 as assessed by immunochemistry [15, 17].

There is so far very little data on PD-L1 expression in cholangiocarcinoma [15, 18]. Interim analysis of a phase 1 trial Keynote028 studying pembrolizumab, an anti-PD-1, in advanced cholangiocarcinoma or gallbladder carcinoma progressing after a first line therapy indicates that PD-L1 was positive in 42% (*n* = 37/89) of patients. Nevertheless, to our knowledge, specific data on the type of PD-L1 expression (tumoral versus immune cells) and differences between intrahepatic, hilar and gallbladder cholangiocarcinoma were not yet communicated. Moreover, profile and incidence of PD-L1 expression may be different in early stage compared to advanced stage. Interestingly, 17% of responses were observed in this interim analysis [19].

This prompted us to investigate PD-L1 immunohistochemical expression in a series of iCCAs and pCCAs, and to correlate our findings with the clinical and pathological features of the tumors and PD-1 expression.

## RESULTS

### Patients and samples

Ninety-nine surgically resected cholangiocarcinomas were included in the study. The median age of the patients was 62.5 years (range 24 to 85) and the sex ratio (male/female) was 2.09. Risk factors of liver disease were identified in 23 patients: hepatitis B virus infection in 8 cases, alcohol in 6, non-alcoholic steatohepatitis in 6, hepatitis C virus infection in 1, primary sclerosing cholangitis in 2, and hemochromatosis in 2. Two patients had multiple risks factors. The main clinical characteristics of the 99 patients are summarized in Table 1. Follow-up data were available in a limited number of cases and could not be used for statistical analysis.

**Table 1:** Main clinical features of the patients and pathological characteristics of the tumors

	iCCA (*n* = 58)	pCCA (*n* = 41)
Age (median, range)	62.5 (33–85)	62 (24–78)
Sex ratio (M/F)	1.9	2.4
**Liver disease**		
None identified	36	40
Hepatitis B virus infection	7	1
Alcohol	6	0
NASH	6	0
Hemochromatosis	2	0
Primary sclerosing cholangitis	2	0
Hepatitis C virus infection	1	0
**Tumor size ± SD (mean in mm)**	73.4 ± 37.4	39.8 ± 39.1
**Tumor differentiation**		
Well/moderately	66% (38)	83% (34)
Poor	34% (20)	17 % (7)
**Vascular invasion**	62% (36)	63% (26)
**Perineural invasion**	33% (19)	88% (36)
**Lymph node metastasis**	47% (15/32)	60% (21/35)
**Density of tumor infiltrating lymphocytes (*n* = 98)**		
High	57% (33)	67% (27/40)
Low	43% (25)	33% (13/40)
**Non tumorous liver fibrosis**		
F0–F1	55% (31/57)	32% (13)
F2–F3	33% (19/57)	66% (27)
F4	12% (7/57)	2% (1)

SD: standard deviation.

### Histological examination

The tumors were intra-hepatic in 59% of cases (*n* = 58) and perihilar in 41% (*n* = 41). Tumor size ranged from 6 to 230 mm (mean 59.5mm) (Table 1). Tumors were well/moderately or poorly differentiated in 73% (*n* = 72) and 27% (*n* = 27) of cases, respectively (Table 1). Invaded surgical margins, vascular invasion, perineural invasion, and lymph node metastases were observed in 41% (*n* = 41), 63% (*n* = 62), 55% (*n* = 55) and 54% (*n* = 36/67) of tumors, respectively. Adjacent non tumoral liver fibrosis could be analyzed in all but one case and was graded as follows: F0-F1 in 45% (*n* = 44), F2-F3 in 47% (*n* = 46) and F4 in 8% (*n* = 8) of cases.

### Immunohistochemistry

Density of CD3-positive intratumoral lymphocytic infiltration was evaluated in 98 cases and scored high in 57% of iCCAs (*n* = 33) and in 67% of pCCAs (*n* = 27/40) (Table 1).

**Figure 1:**
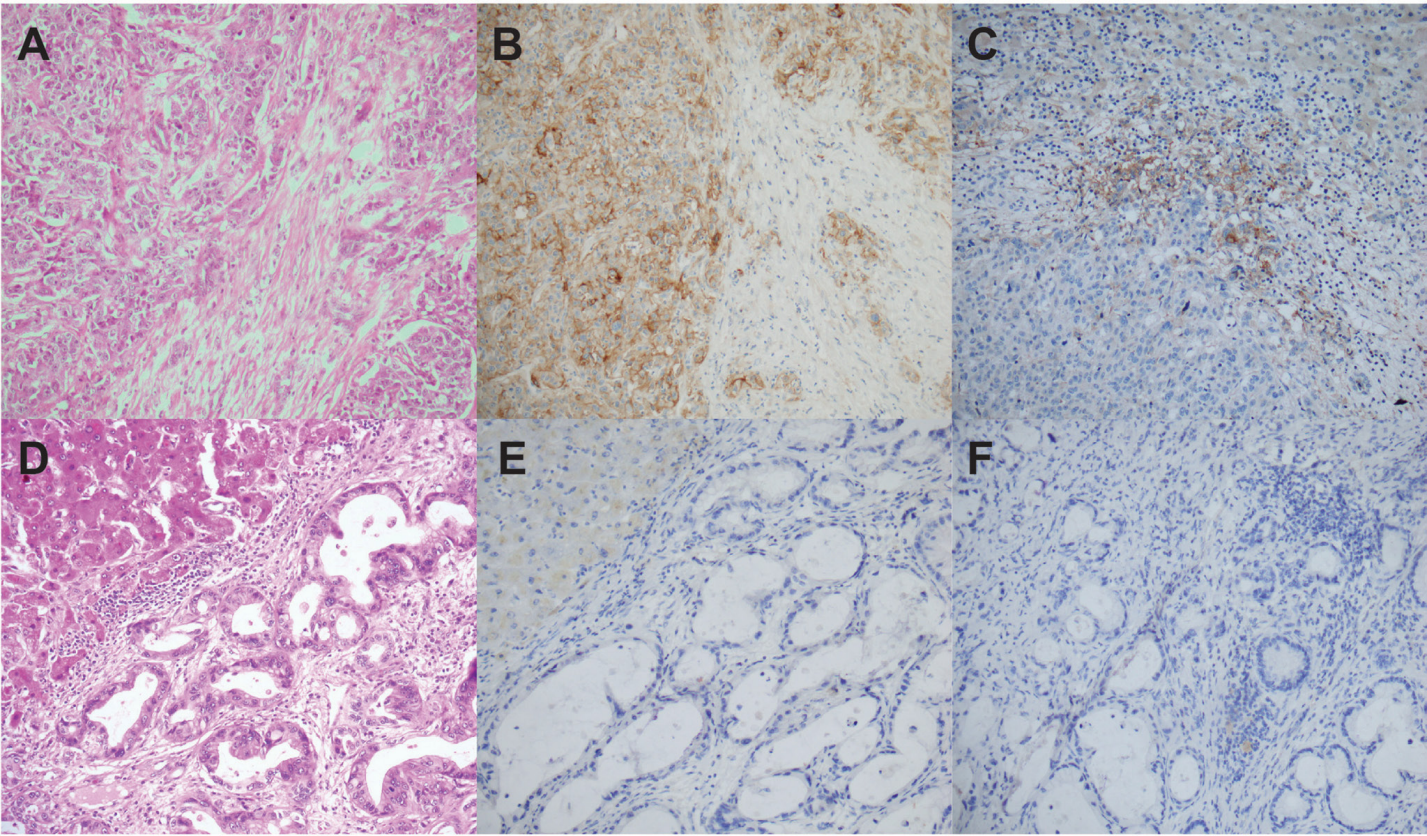
PD-L1 expression in cholangiocarcinoma Poorly differentiated iCCA case showing a massive architectural pattern and no obvious glandular differentiation (hematein-eosin-saffron, X200) (**A**), membranous PD-L1 expression by the vast majority of neoplastic cells in this tumor area (X200) (**B**), and clusters of PD-L1-positive inflammatory cells (X200) (**C**). Well-differentiated iCCA case characterized by a glandular architectural pattern (hematein-eosin-saffron, X200) (**D**), no PD-L1 expression by neoplastic cells (X200) (**E**) or by inflammatory cells (X200) (**F**).

PD-L1 expression by neoplastic cells was observed in a limited number of cases (9%, *n* = 9). PD-L1 positive tumors were intra-hepatic in 5 cases (9% of ICCAs) and perihilar in 4 cases (10% of pCCAs). The percentage of PD-L1 tumor cell expression ranged from 0 to 30%. An almost significant relationship with poor differentiation and high PD-1 expression was observed (*p* = 0.06 and 0.05, respectively) (Table 2 and Figure 1).

**Table 2:** Relationship between PD-L1 expression and the clinico-pathological characteristics of the tumors

	> 5% PD-L1-positive tumor cells	≤ 5% PD-L1-positive tumor cells	*p*-value	PD-L1-positive inflammatory cell aggregates (mean ± SD)	*p*-value
**Age**					
> 62 yrs	3	46	0.49	5.7 ± 10.6	0.88
≤ 62 yrs	6	44		7.5 ± 16.6	
**Sex**					
Male	7	60	0.71	7.3 ± 14.8	0.51
Female	2	30		5.2 ± 12	
**Tumor location**					
Intrahepatic	5	53	1	8.5 ± 16.7	0.14
Perihilar	4	37		3.9 ± 8.3	
Tumor size					
> 50 mm	3	46	0.49	8.3 ± 16.6	0.37
≤ 50 mm	6	44		5.1 ± 10.9	
**Tumor differentiation**					
Well/moderately	4	68	0.06	9.5 ± 17.4	0.20
Poor	5	22		5.5 ± 12.4	
**Density of tumor-infiltrating lymphocytes (*n* = 98)**					
High	9	51	0.01	10.0 ± 16.9	0.005
Low	0	38		1.5 ± 3.3	
**Vascular invasion**					
Present	5	57	0.72	7.7 ± 9	0.93
Absent	4	33		4.9 ± 16.2	
**Perineural invasion**					
Present	6	49	0.73	6.7 ± 14	0.29
Absent	3	41		6.6 ± 14	
**Lymph node metastasis**					
Present	4	32	1	8.0 ± 15	0.58
Absent	3	28		4.1 ± 13.4	
**PD-1 expression**					
High	4	14	0.05	19.7 ± 22.2	< 0.0001
Low	5	76		3.8 ± 9.3	

SD: standard deviation.

Aggregates of PD-L1-positive inflammatory cells were identified in 46% (*n* = 46) of cases, including 31 iCCAs and 15 pCCAs (Figure 1). The number of PD-L1-positive aggregates per 10 HPFs ranged from 0 to 67 (mean: 6.6 ± 14).

PD-L1 expression by either neoplastic or inflammatory cells was associated with high density of TILs (*p* = 0.01 and *p* = 0.005, respectively) (Table 2).

**Figure 2:**
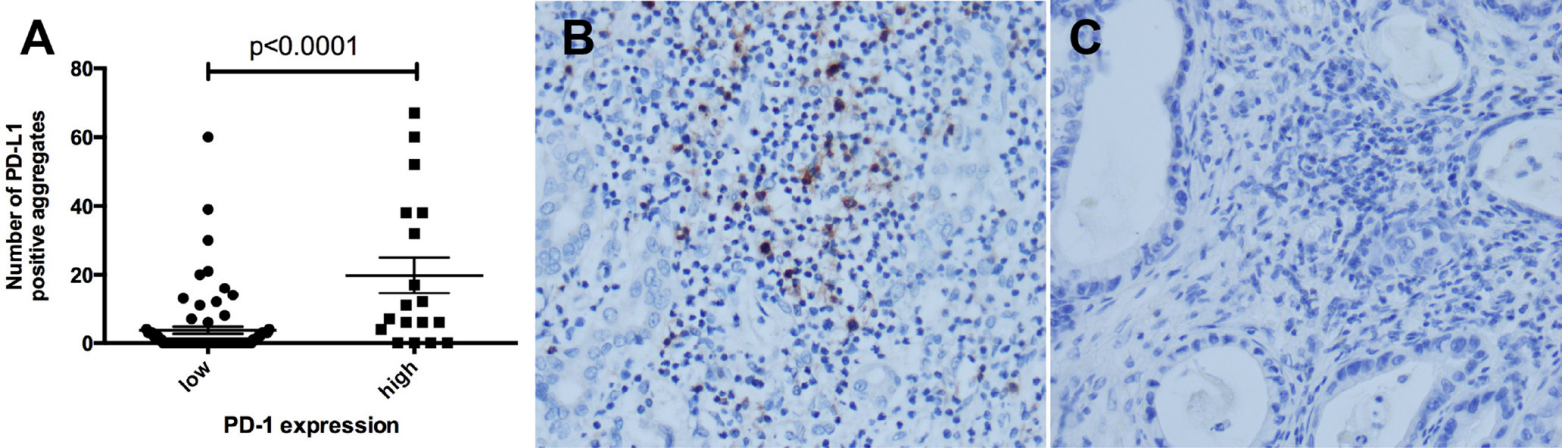
PD-L1 expression by inflammatory cells is significantly higher in PD-1 high tumors Number of PD-L1 positive inflammatory cell aggregates in tumors with low and high PD-1 expression (*p* < 0.0001) (**A**). Representative micrographs of high (**B**) and low (**C**) PD-1 expression (X400).

The number of PD-L1 positive aggregates of inflammatory cells was also higher in tumors with high PD-1 expression (*p* < 0.0001) (Figure 2).

## DISCUSSION

Cholangiocarcinoma is a highly aggressive neoplasm characterized by the lack of effective therapy and a dismal prognosis. Recent clinical trials using PD-L1/PD-1 immune checkpoint blocking agents have shown efficacy in various malignancies, with responses strongly associated to PD-L1 expression, as assessed by immunochemistry [15]. In the present study, we evaluated the immunohistochemical expression of PD-L1 in a series of iCCAs and pCCAs, and compared our findings with the clinicopathological features of the tumors -including TILs density- and PD-1 expression.

We first observed that PD-L1 was mainly expressed by intratumoral immune cells in both iCCAs and pCCAs, with very few cases expressing PD-L1 in neoplastic cells. Sabbatino et al. studied PD-L1 and PD-1 expression in a series of 27 iCCAs and observed tumoral expression of PD-L1 in a higher proportion of cases than ourselves (30%) [20]. However, the authors specified that tumors with “rare” staining were considered positive. This may suggest that they included cases with less than 5% of tumor cell staining. Interestingly, in a recent meta-analysis of 27 clinical trials using PD-1/PD-L1 blocking agents, Gandini et al. showed that clinical trials using a 5% positivity cut-off for PD-L1 expression in tumor cells correlated with objective response, but the relationship was lost when a 1% positivity cut-off was used [21].

It is unknown whether PD-L1 expression by immune cells has the same predictive value on the response to therapy as its expression by tumor cells. It is now well established that intratumoral inflammatory cells may also contribute to T cell exhaustion and anti-tumor immunity suppression through PD-L1 expression [22, 23]. The relationship between PD-L1 and PD-1 expression reported here also supports the existence of a functional PD-L1/PD-1 immune checkpoint in cholangiocarcinoma. Moreover, as already reported in colorectal carcinoma, we observed enhanced PD-L1 expression by the microenvironment in cholangiocarcinomas with dense lymphocytic infiltrates, suggesting that these tumors stimulate the immune system and rely on the PD-L1/PD-1 pathway to escape antitumor immunity [24, 25].

Altogether, this study shows that PD-L1 is mainly expressed by intratumoral inflammatory cells in both iCCAs and pCCAs, principally in tumors with dense intratumoral lymphocytic infiltration. Our results suggest that CCAs with dense intratumoral lymphocytic infiltration could be good candidates for PD-L1/PD-1 blocking agents.

## MATERIALS AND METHODS

### Patient population and specimen collection

Patients included in the study underwent surgical resection for iCCA or pCCA at Henri Mondor Hospital, Créteil, France, between 1998 and 2015. Patients with combined hepatocellular-cholangiocarcinoma or with prior chemotherapy treatment were excluded.

Age, gender and risk factors of chronic liver disease were systematically recorded. Histological slides were retrieved from the Pathology Department archives. The study was approved by the Local Ethics Committee of Saint-Louis Hospital, ‘Ile de France IV’ (IRB no. 00003835).

### Histopathological analysis

Three pathologists (J.F, J.A, J.C) reviewed all of the available hematein-eosin-saffron stained slides for each case. Diagnosis was confirmed according to the WHO classification system [26]. Each tumor specimen was analyzed for the following pathological parameters: tumor size, location (perihilar or intrahepatic) and degree of differentiation, perineural and vascular invasion, lymph node metastasis, and surgical resection margins. Non tumoral adjacent liver was evaluated for the degree of fibrosis according to the METAVIR scoring system [27].

One representative paraffin-embedded block representing the tumor area with the highest density of immune infiltration was selected. Although some representative slides included the tumor border, all immunohistochemistry stainings were evaluated strictly within the tumor, not at the tumor front.

### Evaluation of intratumoral lymphocytic infiltration density

Density of intratumoral infiltrating lymphocytes was assessed on the representative tumor slide by immunohistochemistry using a polyclonal rabbit anti-human CD3 antibody (DAKO A0452). After examination of a training set comprising 35 tumors, CD3 expression was scored in the densest infiltrative areas within the tumor, by counting the number of high power fields (HPFs) (X400, 0.26mm^2^) with greater than 100 CD3-stained TILs. Tumors with less than 10 HPFs comprising at least 100 CD3+ TILs were scored “low” and those with 10 or more were scored “high” for intratumoral lymphocytic infiltration density. CD3 staining could not be performed in one case due to block exhaustion.

### PD-L1 and PD-1 expression by immunohistochemistry

All immunostainings were performed on 4 μm-thick whole sections of the representative tumor block, to avoid sampling heterogeneity inherent to tissue-microarray techniques. After deparaffinization and rehydration, antigen retrieval was performed in BondMax Epitope Retrieval buffers (Leica Biosystems, Nussloch, Germany) at pH6 (PD-1) or pH9 (PD-L1) with the primary antibodies Abcam AB52587 and Cell Signaling E1L3N, respectively. Immunodetection was performed with a biotin-conjugated secondary antibody (Vision BioSystems DS9713, Menarini, Florence, Italy) followed by peroxidase-labeled streptavidin and with diaminobenzidine chromogen as the substrate (Vision BioSystems DAB, Menarini). Slides were processed on an automated immunostainer (Leica Bondmax) according to the manufacturer's instructions. Anti-PD-L1 and anti-PD-1 antibodies were verified and calibrated on appropriate external positive control tissues, with known constitutive positivity. Placenta was used for PD-L1 (strong syncitiotrophoblastic cell positivity) and benign lymph node tissue for PD-1 (strong positivity of follicular helper T-cells).

PD-L1 expression was assessed in both tumor cells and tumor-infiltrating inflammatory cells. Tumor cells were considered PD-L1-positive in the presence of strong membranous staining (easily identified at X100) in at least 5% of tumor cells [28]. Inflammatory cells (macrophages and/or lymphocytes) expressing PD-L1 formed small aggregates interspersed within the tumor area, as previously reported in other malignancies [16, 29, 30]. We thus decided to assess PD-L1 expression in inflammatory cells by counting the number of positive clusters in 10 high-magnification fields (X400, 0.26mm^2^).

PD-1 was only expressed by lymphocytes, and its expression, after examination of a training set comprising 35 tumors, was assessed in 10 HPFs in intratumoral areas with the highest density of stained cells. Tumors with at least 5 HPFs comprising 20 or more PD-1 stained lymphocytes were scored “PD-1 high”, those with less were scored “PD-1 low”.

### Statistical analysis

Statistical analysis was performed using Graphpad Prism 6 Software. Continuous variables and proportions were compared using Mann-Whitney and Fisher exact tests, respectively. *p* values < 0.05 were considered statistically significant.
